# Addressing the healthcare needs of older Lesbian, Gay, Bisexual, and Transgender patients in medical school curricula: a call to action

**DOI:** 10.1080/10872981.2017.1320933

**Published:** 2017-05-04

**Authors:** Sophie M. Cannon, Vipul Shukla, Allison A. Vanderbilt

**Affiliations:** ^a^ College of Medicine and Life Sciences, University of Toledo, Toledo, OH, USA; ^b^ LGBT Health Policy and Practice Graduate Program, George Washington University, Washington, DC, USA; ^c^ Department of Family Medicine, College of Medicine and Life Sciences, University of Toledo, Toledo, OH, USA

**Keywords:** Aging, LGBT, curricula reform, healthcare disparities, medical school

## Abstract

Medical students matriculating in the coming years will be faced with treating an expansive increase in the population of older lesbian, gay, bisexual, and transgender (LGBT) patients. While these patients face healthcare concerns similar to their non-LGBT aging peers, the older LGBT community has distinct healthcare needs and faces well-documented healthcare disparities. In order to reduce these healthcare barriers, medical school curricula must prepare and educate future physicians to treat this population while providing high quality, culturally-competent care. This article addresses some of the unique healthcare needs of the aging LGBT population with an emphasis on social concerns and healthcare disparities. It provides additional curricular recommendations to aid in the progressive augmentation of medical school curricula.

**Abbreviations:** Liaison Committee on Medical Education (LCME); LGBT: Lesbian, gay, bisexual, transgender

## Introduction

In the year 2000, 1 to 2.8 million lesbian, gay, bisexual, or transgender (LGBT) adults aged 65 and older were living in the United States [1,2]. By the year 2030, this number is estimated to reach between 2 and 6 million [1,2]. Medical students matriculating in the coming years will likely be faced with treating this expansive increase in the population of older LGBT patients. While these patients face healthcare concerns similar to their non-LGBT aging peers, the older LGBT community has distinct healthcare needs and faces well-documented healthcare disparities [3]. As such, medical schools must prepare and educate future physicians to treat the unique healthcare requirements of this population while providing culturally-competent care.

Recently, the Association of American Medical Colleges (AAMC) published a 300+ page resource for medical educators outlining the implementation of curricular and institutional climate changes to improve the healthcare of LGBT and gender nonconforming patients [4]. The push for improved cultural competence and increased education for physicians has been remarkably encouraging. However, particular needs of aging LGBT patients are less documented among current medical education standards [5]. While the AAMC resource lays out some concerns, this call to action addresses a few of the unique healthcare needs of the aging LGBT population, with an emphasis on social concerns, healthcare disparities, and cultural competence. These issues parallel Liaison Committee on Medical Education (LCME) accreditation standards 7.2 and 7.6 [5], and emphasize the growing need for additional cultural competence training focused on the older LGBT patient population.

## Healthcare disparities

Older LGBT adults experience significant health disparities related to aging [6]. In a population-based study analyzing data of healthcare outcomes and access to care, older LGB adults had an overall higher risk of disability, poor mental health, smoking, and increased alcohol consumption than did heterosexuals [6]. Older lesbian women have a higher risk of developing metabolic syndromes and cardiovascular disease [1,3]. In conjunction with this increased risk, they are less likely to be provided preventative healthcare screenings such as mammograms [1], highlighting the crucial need for increased education of healthcare providers. Older transgender adults are at significantly higher risk of poor physical health, disability, depression, and perceived stress compared to non-transgender patients [6].

## Social concerns

Aging LGBT individuals experience a wide array of physical and psychological healthcare concerns synonymous with their heterosexual, binary, or gender-conforming peers [7]. However, LGBT older adults are more than twice as likely to live alone and two times less likely to be partnered [1,8]. As older LGBT adults are more likely to be single or without children, they are consequently much more likely to have a ‘chosen family,’ or a group of people to whom one is emotionally close and considers ‘family,’ even though one might not be biologically or legally related [1,9]. These chosen support networks can be threatened by aging and illness [1]. Healthcare providers and students must be alert to the importance of nonrelatives as a source of support for this community.

With these ‘chosen families’ comes significant differences in caregiving and social support networks in the older LGBT community. While studies of the general US population indicate that between 25–44% of caregivers are male, within the LGBT community there is an even percent of male and female LGBT caregivers [10]. Thus suggesting that gay, bisexual, and transgender men may be providing care to parents, partners, and friends more often than non-LBGT men. While 75% of respondents expected to eventually become caregivers to someone else, 20% were unsure who would eventually care for them [10]. For individuals without partners, 33% of LGBT patients are unsure about care [10]. The discrepancy illustrates worried views of an LGBT patient population that faces unknown future support systems.

Considerations regarding ‘chosen families’ and unique caregiving responsibilities impact decisions related to retirement and end-of-life care. Completion of a living will or durable power of attorney is approximately 29% for both LGBT adults and non-LGBT adults [11]. However, for older LGBT individuals who have a ‘chosen family’ or are partnered, these considerations take on additional importance [10]. Unless an LGBT adult is married or has expansive legal arrangements, most states give priority to opposite-sex spouses and blood relatives for medical and long-term care decision-making and visitation [12]. In accordance with LCME Standard 7.2, which highlights end-of-life care and health-related impact on patients of behavioral and socioeconomic factors [5], it is imperative that current and future physicians be sensitive and knowledgeable of these challenges.

## Advancing medical education

In accordance with LCME accreditation Standard 7.6 emphasizing culturally competent care and development of solutions for healthcare disparities [5], students should be given greater instruction on the social and healthcare-related experiences of older LGBT patients. There are many opportunities for infusion of LGBT eldercare content into current standardized medical school curricula in a longitudinal manner. At the University of California, San Francisco (UCSF), medical students associated with UCSF’s LGBT Resource Center developed a map of existing curricular offerings related to LGBT healthcare, and identified areas of improvement [13,14]. Students systematically contacted teaching faculty and implemented LGBT healthcare content within the established curricula [13,14]. Similar approaches could be utilized to address specific gaps in knowledge associated with LGBT eldercare. For example, an immunology course teaching about HIV might include information that over half of those living with HIV will be over the age of 50 in the coming years [15]. Evidence that practicing physicians are less likely to suspect STDs and new-onset HIV in older LGBT patients [7] could also be discussed within this setting. When students learn about the endocrine system, they might be informed that as transgender individuals age, they are more likely to encounter health issues related to their biological sex, which can lead to additional stressors in coping with diseases associated with a sex they do not identify with [7]. Here, students might be educated about appropriate ways to tailor preventative care to biological sex in this population, while being cognizant of psychological and sociological factors that may impact this care [7]. Normalizing the healthcare needs of older LGBT patients and increasing clinical awareness of specific healthcare concerns is important in shaping well-rounded, culturally competent student doctors.

In addition to addressing concerns related to health disparities, medical students must be advised about the unique support systems and potential stressors in order to provide improved, culturally competent care to their future patients. This can be accomplished by increasing exposure of students to older LGBT adults by utilizing a panel of local community members. This panel could be modeled from The Gay and Grey Program (GGP)’s trainings given by older LGBT adult volunteers to students and professionals in Portland, Oregon [16]. Overall goals of GGP trainings include educating participants, improving services, and reducing barriers for older LGBT adults. The GGP panel consists of a video depicting information about financial discrimination, discriminatory policies in skilled nursing facilities, and issues related to caregiving [16]. Personal narratives by panel members and interactive educational exercises are also presented [16]. These experiences helped raise awareness and understanding about issues faced by older LGBT adults [16]. A large percentage of participants indicated these trainings helped them to reflect upon personal biases and assumptions toward LGBT adults [16].

In addition, complex end-of-life care decisions and the legal experiences of LGBT patients with ‘chosen families’ could be addressed in medical school ethics courses by utilizing a Problem Based Learning (PBL) approach. Unlike traditional passive learning with professor-designed didactic lectures, PBL involves active learning with dynamic interactions between learners and teachers [17]. Students are presented with a challenging problem, and are guided in brainstorming practical solutions with real-world implications [17]. When compared to traditional lectures, PBL implementation results in greater increases in critical thinking [18] and enhances clinical reasoning competency [17]. Within our model of older LGBT patient care, students would be presented with specific LGBT eldercare clinical cases. This activity would emphasize understanding of ‘chosen families’, complex end-of-life patient issues, and the development of solutions. Students would be directly tasked with thinking critically about their own roles in culturally competent care, considering questions such as, ‘What legal barriers might impact a patient with an extensive “chosen family”? How might a healthcare provider mitigate these issues?’ Providing a structured format for medical students to reflect on these complex social challenges lays an educational framework for more adept and culturally competent care.

## Conclusion

In order to educate a new generation of physicians equipped to provide culturally competent care, specific gaps in knowledge must be assessed. There are well-documented gaps in the cultural competency of LGBT older adult patients among health and social service providers [19]. Gaps in knowledge and education of healthcare providers appear to correlate with perceptions shared by the older LGBT patient population. More than a quarter of older LGBT adults report great concern about discrimination as they age [10]. Only ~50% expressed confidence that healthcare providers would ‘treat them with dignity and respect’ [10]. Given that over 120 000 LGBT seniors will be living in nursing homes by 2030 [20], future physicians must be aware that real or anticipated fear of discrimination exists, and may lead to fear of disclosure and additional unmet healthcare needs.

Societal problems of perceived discrimination, coupled with a lack of adequate training for future physicians, may contribute to unmet healthcare needs of the older LGBT patient population. Increased cultural competency related to physician-patient relationships must be addressed through progressive augmentation of medical school curriculum. As current and future healthcare providers, we must see that the needs of older LGBT adults are adequately addressed in medical education in order to ensure well-rounded, culturally competent care.
